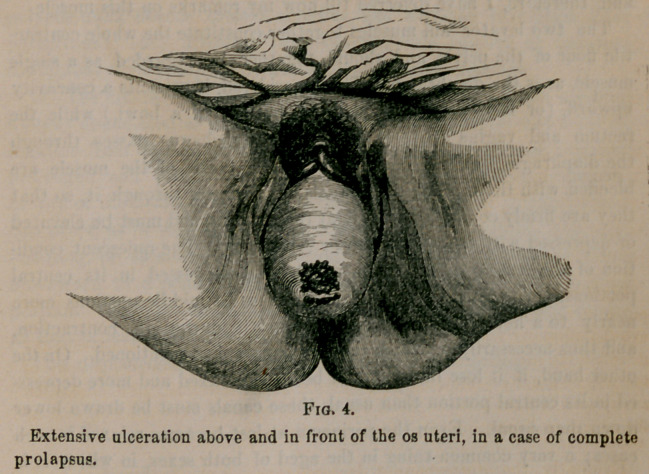# Lectures on Displacements of the Uterus

**Published:** 1860-05

**Authors:** E. R. Peaslee

**Affiliations:** Prof. of Obstetics and Diseases of Women and Children in the New York Medical College


*Lectures on Displacements of the Uterus.* By E. R. Peaslee, M.D., LL.D., Prof. of Obstetrics and Diseases of Women and Children in the New York Medical College.

(Continued from last No. of the Monthly.)

LECTURE III.

Gentlemen—The displacements of the non-gravid uterus may, for all practical purposes, be arranged under two classes:

1st. Displacements of the whole uterus *downward*—including prolapsus in its various degrees, and inversion. The latter usually occurs, however, in connection with parturition.

2d. Displacements of the whole uterus, or of its body alone, either *forward* or *backward*—anteversion or anteflexion, and retroversion or retroflexion.

I might also add displacement of the whole uterus upward—*elevatio uteri;* and lateral displacements of the body, or of the whole organ. But the former, if uncomplicated, requires no treatment; while the latter are mere complications with the anterior and posterior displacements, or are produced by the direct pressure of abnormal developments within the pelvis, and will be disposed of in connection with the second class of displacements.

Pretermitting inversion of the uterus, for the present, since its most common method of causation separates it, practically, from the others—I shall consider the displacements in the following order:

| | | |
|---|---|---|
| 1. | Prolapsus.<br>Inversion. (4.) | Displacements of the whole uterus downward. |
| 2. | Anteversion.<br>Anteflexion. | Displacements of the whole uterus, or of its body only—forward. |

3. { Retroversion. } Displacements of the whole uterus, or of its body
{ Retroflexion. } only—backward.

You will, therefore, entirely isolate prolapsus uteri from the other class of displacements. For, though both classes may present the same local symptoms, as enumerated in my first lecture, and may also ultimately induce the same general symptoms, still the local treatment of the two classes is, as a general rule, conducted on different principles. The local treatment is, however, in both classes, indispensable, and especially determines the curative result. For while it often fails without the general treatment, the latter very rarely succeeds without the local; and the local in many cases succeeds alone. It is, therefore, more especially in aid of the local treatment that the general is resorted to.

But before commencing with prolapsus, I will, in order to avoid repetition, speak of the general or constitutional symptoms common to all the displacements, as I have before spoken of their common local symptoms. And we find that they affect the nervous, the digestive, and the circulatory systems.*

A. *Symptoms affecting the nervous system.*—It is generally understood that those suffering from uterine diseases are the most nervous of all patients. All understand the many symptoms developed by the uterus during pregnancy; and those of diseases of the same organ are neither less numerous nor essentially dissimilar. All the functions of both the cerebro-spinal and the sympathetic nervous system are liable to become deranged. Hence, we find abnormal sensations, impairments of the motor power, and morbid states of the mental faculties, as affections of the former; together with derangements of all the organic functions. It will be remembered that all the symptoms of the displacements which I am about to enumerate may occur in other uterine affections also.

1. As morbid *sensations* due to uterine displacements, I refer to the local pains and other peculiar sensations in various parts of the body, enumerated in my first lecture. But there are others of which I must here take account. Irritation of the bladder, and of the rectum; an exquisitely painful sensitiveness of the vagina; and a distressing pain referred to the point of the os coccygis—are not uncommon symptoms. Also may be added, pain along the crest of the ilium, or above the pubes; a feeling of *malaise* in the region of the ovaries, (especially of the left;) a pain under the edge of the ribs, more frequently on the left side, and suggesting to the patient the idea of heart-disease; or

---

* Dr. Peebles, in *Am. Journ. of Med. Sciences*, Jan. 1853, p. 41–8.

seeming, if on the right side, to indicate some hepatic derangement; pain, tenderness, or swelling of one or both mammæ; pain on pressure over some of the spinous processes of the vertebral column; and an exaggerated sensibility of the surface of the abdomen, or even of the whole body. All the preceding come under the head of reflex pain, before alluded to. I have, in three instances, seen this acute sensibility of the whole abdominal surface developed (to terminate spontaneously in a few hours) by the introduction of the uterine sound. Another symptom, almost pathognomonic, of uterine affections, is the "uterine headache," referred to the top of the head, usually extending over a circular or oval surface, and which is relieved by pressure. Sometimes, however, instead of pain, a "crazy feeling," a sensation of cold or heat, or a numbness, is complained of; or the surface is tender on pressure, or found to be preternaturally hot. Sometimes a neuralgic pain extends over the entire scalp. These sensations in the head are sometimes relieved the instant the uterus is replaced, again to return at once, if it relapses into its displaced condition. The same is also true of many others of the symptoms I have mentioned; and especially those affecting the back, the groins, and the thighs.

On the other hand, numbness or sensations of cold, affecting any part of the body, may frequently be observed; especially numbness in the groin and anterior surface of the thighs; and coldness of the hands and feet are among the most frequently occurring symptoms.

2. Under derangements of the *motor* function we have every variety of deranged muscular action, whether of debility, or of excessive or irregular action. Hence, a feeling of languor, affecting the whole muscular system, or the back and lower extremities alone, and impairing the ability to walk. Various forms of spasmodic affections are also to be added, of which I specify the following: A loud, dry, spasmodic cough, distinguished by the suddenness of its appearance and its disappearance; various modifications of the respiratory movements; spasmodic affections of various sets of muscles, and not seldom of those of the back of the neck, causing the head to be drawn backward. In a word, all those irregular actions which we see developed in *hysteria*, belong here; this disease, in its ever-varying phases, being one of the most common effects of uterine displacements. Very often we find spasmodic twitching in the groins, the leg, the eyelid, and the abdominal muscles.* Palpitation of the heart is also a very common symptom.

3. But not the least important symptoms of uterine displacement

---

* Dr. Peebles, as above, p. 45.

are those affecting the patient's *mental* condition. We often find her *morale* completely changed. She has, perhaps, become impatient, selfish, is despondent, and avoids society; is irritable, and perhaps thrown, by the least opposition to her wishes, into great excitement, or even into an hysterical paroxysm. The intellectual faculties also suffer. Debilitated by want of exercise, and prolonged confinement within doors, such patients lose their powers of volition and intellectual exertion, and become a prey to their morbid fancies and painful sensations of every kind; a "condition sometimes ending in insanity, and often resulting in a state of mind but little short of it."—*Peebles.* This result, moreover, is not seldom precipitated by the unsympathizing conduct of the husband or friends of the patient; who ignorantly assume that these symptoms are due to a merely imaginary disorder, that the poor victim is merely "nervous."

B. *The symptoms affecting the organic functions* are also very numerous. Some of the symptoms already mentioned are, indeed, often dependent on previous derangements of this class of functions.

1. Much derangement of the digestive and secretive functions occurs in the course of these displacements. Loss of appetite, or a depraved condition of it; constipation, (often due to direct mechanical action, as in cases of retroflexion;) tympanites from accumulation of gas in the intestines; torpidity of the liver; and great variableness in the amount of urine secreted—may be mentioned here. Sometimes anything taken into the stomach becomes so excessively acid as to affect the teeth, and is rejected in that state. Dr. Peebles regards this condition as peculiar to uterine derangements. Diarrhœa exists but rarely. From all these causes, the patient becomes thin and sallow, and prematurely old.

2. Finally, anæmia and all its effects ensue from the causes of malnutrition, just mentioned, and the circulatory system, of course, also suffers; this condition also reacting on the functions of the cerebro-spinal nervous system, as before explained. The action of the heart becomes irregular and feeble, or greatly excited—symptoms giving the patient great distress and anxiety; and the minute vessels lose their power of controlling the circulation. Hence those sudden flushes of the face without assignable cause, which so annoy this class of patients. I have, in several instances, seen a permanent blush, in such cases, of the whole neck and upper part of the chest; and the application of a sinapism to the surface sometimes produces an unexpectedly severe result, even endangering sloughing of the skin.

I have spoken thus at length of the constitutional symptoms of uterine displacements, since they alone are often treated to the entire neglect of their cause, and with the hope of thus putting you upon your guard against the commission of such an error.

### I.—Prolapse of the Uterus.

This displacement is also variously termed descensus uteri, prolapsus, and procidentia; and by patients themselves, falling of the womb. It is a displacement downward, without inversion, of the whole uterus.

Dr. Meigs maintains* that "prolapsus uteri is an affection of the vagina, and not of the womb itself; cure that canal, and you will find the womb cured also." If merely intended to inculcate the practical fact that prolapsus generally requires mechanical treatment applied *per vaginam*, this assertion has an appearance of correctness; but as a definition of prolapsus, or as a literal expression of fact, even in regard to treatment, it is very objectionable, and quite untenable.

1. It does not designate the particular affection of the vagina which prolapsus is, and therefore does not define the latter at all. It must refer to the shortening, or inversion, or both, of the vagina; but these are not prolapsus uteri. If so, this expression would be quite superfluous.

2. Dr. Meigs' assertion identifies prolapsus with its effects. The uterus can be displaced downward only by falling into the vagina, and descending in the course of this canal. And since the vagina is attached above, around the cervix, as before explained, that portion must descend with the cervix uteri, and inversion of the vagina from above downward must ensue. Inversion of the vagina is, therefore, a direct and necessary effect of prolapsus uteri, and this inversion, of course, produces a virtual shortening of the vagina as a secondary effect of prolapsus. Inversion and consequent shortening of the vagina are therefore not prolapsus, any more than deformity of a limb is fracture.

3. This proposition also exemplifies the *prima facie* inconsistency of asserting that a particular condition of one organ is an entirely different condition of *another* organ; as if I should say that derangement of the stomach is inflammation of the eyes.

4. Finally, the assertion that the prolapsus is cured by curing the vagina, is not strictly correct in any case of prolapsus, and is the very reverse of correct in most cases. For, in very many cases, the cure is effected by treatment applied to the uterus directly, and not to the vagina at all; while it is directly applied to, and for the sake of cur-

* Woman and her Diseases, p. 200.

ing the vagina, only in the proportionally very few instances in which a disease (as relaxation) of the vagina was the original cause of the prolapsus. A pessary, even, is usually applied, not to cure the vagina, but to keep the uterus from descending into the vagina—*i. e*, to cure the prolapsus, and thus allow the vagina to resume its normal condition.

I have made these remarks to guard you against the idea that the treatment of prolapsus should be directed exclusively to the vagina; which the assertion I have objected to might lead you to adopt. For we shall see that it should be instituted far more to remove the causes of prolapsus, than to cure its effects. The following proposition, therefore, though liable to most of the objections I have raised to the one I have quoted, is much to be preferred to it: "Inversion, with consequent shortening, of the vagina, is prolapsus uteri; cure the prolapsus, and the vagina will be cured as a matter of course."

Prolapsus uteri may, therefore, be defined to be, *a descent, without inversion, of the uterus into, or through the whole of, the vagina; necessarily producing a proportionate inversion and consequent shortening of that canal.*

It is the most common form of uterine displacement. It may occur in all ranks and conditions of society, in the married and the unmarried, and at all ages. Dr. Alexander Monroe speaks of an instance in a child but three years of age. It, however, occurs more rarely in the virgin state, unless from some malformation, than the second class of displacements. It is the most common in those who have borne children, and quite often occurs after rupture of the perineum.

The average distance of the os uteri from the ostium externum is (Lect. 1) $3\frac{1}{2}$ inches; though, in some cases, it is not more than 2 inches. Still, so short a vagina does not at all necessitate prolapsus, though it may well be regarded as a predisposing cause. The uterus may also descend to any extent into the vagina, until it is at last completely extruded through the ostium externum, and the vagina is completely inverted.

Three degrees of prolapsus will, for practical purposes, be recognized. In the

*First*—the cervix uteri falls only so as to rest on the posterior wall of the vagina; *i. e.*, through the upper third or less of the vagina.

*Second Degree*—the cervix uteri rests on the internal surface of the perineum, or descends to the ostium externum.

*Third Degree*—the uterus is entirely extruded, and the vagina is, consequently, completely inverted.

Various terms have been applied to these degrees of prolapsus, and the following arrangement may prevent confusion:

*First Degree.*—Incipient prolapsus, (Churchill;) delapsion, (Davis;) relaxation.

*Second Degree.*—Procidentia, (Churchill;) prolapsion, (Davis;) semi-prolapse, (Boivin;) delapsus, (Kulm.)

*Third Degree.*—Complete prolapse, (Churchill;) procidentia, (Davis.) Nanche and some other French writers include the first two degrees of prolapsus under the term "relachement," while the third or complete prolapse is called "descente."

The relations just specified of the parts in complete prolapsus are shown by the following cut, which is somewhat improved from Churchill's work on the Diseases of Women.

FIG. 3.

Right half of the bisected pelvis in case of complete prolapsus. 1, symphysis pubis; 2, fundus of the bladder, the base being drawn down to form part of the external tumor; 3, rectum; 4, os uteri; 5, urethra, much dilated, and showing the direction the catheter must take; 6, fundus uteri; 7, cavity behind the body of the uterus, usually containing convolutions of the intestines, as does also the cavity seen in front between the uterus and the bladder. The anterior wall of the rectum also sometimes falls down into the cavity, 7.

Evidently, the uterus cannot descend through the curve of the vagina without continually changing the inclination of its long diameter to the superior plane of the pelvis. The cervix must come farther and farther forward as it descends, and the fundus uteri fall proportionally backward; and thus a degree of retroversion necessarily attends on the first and second degrees of prolapsus. Some maintain that this movement of the fundus backward necessarily implies an elongation of the round ligaments; and therefore, that relaxation of these ligaments is indispensable to, if not the main cause of, prolapsus. Since, however, the cervix comes forward in proportion as the fundus inclines backward, no essential elongation of the round ligaments is necessitated, it would seem, till the third degree is reached, when they become stretched by the weight which is brought to bear upon them. Their relaxation and elongation is, therefore, quite as often an effect as a cause of prolapsus. A previous elongation would doubtless predispose to prolapsus, but far more to retroversion or retroflexion.

In the third stage of prolapsus, the entire uterus being extruded through the vulva, the vagina, being completely inverted, will be seen extending *upward* from the cervix uteri to the vulva, and inclosing a conical mass, which consists of the uterus, a part of the bladder and of the rectum, and generally also of some convolutions of intestines. The abnormal position of the bladder often leads to retention of urine; and to relieve it a male catheter must be used, with its convexity presenting downward.

FIG. 4.

Extensive ulceration above and in front of the os uteri, in a case of complete prolapsus.

In the third degree of prolapsus, ulcerations also very frequently occur from the contact of the clothing, on the lower portion of the extruded mass; and sometimes the prolapsus is irreducible. Fig. 4 gives a very good idea of the case of the Mexican woman whom you will recollect as having recently come before you. It also shows the ordinary form and relative size of the mass extruded.

The *causes* of prolapsus are quite numerous. Some would render this a very simple matter by referring prolapsus to a relaxed state of the vagina as its principal cause. This state of the vagina does not, however, induce prolapsus, if the agency of the other natural supports of the uterus is still unimpaired. I must refer you to the account of these supports given in my first lecture, and also give some account of the relations of the levator ani muscle, before we can arrive at an explicit understanding of this subject.

The *direct* supports of the uterus are the broad, the round, and the utero-rectal ligaments; to which add the posterior wall of the bladder, and the vagina also, provided it maintains its own tone and normal position. Its *indirect* supports are the rectum, the levator ani muscle, and the perineum; *i. e.*, if the direct supports fail, these latter may arrest, or even prevent displacement; and on the other hand, these failing to maintain their normal relations, such failure may predispose to, or even produce, displacements. It is mainly prolapsus which is produced by the failure of the indirect supports of the levator ani, and, therefore, I have deferred till now my remarks on this muscle.

The two levator ani muscles together constitute the whole contractile floor of the pelvis, and should together be regarded as a single muscle, as is the diaphragm. Thus considered, it presents a concavity upward, (or it is depressed like the bottom of a bowl,) while the rectum and vagina traverse it, (as the œsophagus passes through the diaphragm,) on the middle line. The fibres of the muscle are blended with those of the two canals as they pass through it, so that they are firmly connected with it at these points, and must be elevated or depressed with its contraction or relaxation. The quiescent condition of the muscle being that in which it is depressed in its central portions, (or is concave upwards,) it raises its middle portions more nearly to a horizontal position in the pelvis during its contraction, and thus necessarily elevates the two canals just mentioned. On the other hand, if it lose its tone, and becomes relaxed and more depressed in its central portion than usual, these canals must be drawn lower down than usual. Even the perineum at last becomes relaxed in such cases; a very common thing in the aged of both sexes, in whom it is seen, on an external examination, bulging downward. In these circumstances the antagonism between the diaphragm at the upper limit of the abdomen, and the levator ani, at the lower, is destroyed; for while, normally, the levator reacted against the diaphragm, and returned to the latter, by its contractile force, the pressure received from the mass of abdominal contents from the contraction of the diaphragm, it has now lost its power of resistance, and the abdominal viscera are now crowded downward by the diaphragm upon the pelvic, and the latter upon the floor of the pelvis, without hindrance. It is, therefore, very easy to perceive how loss of power in the levator ani may predispose to, or may even produce, prolapsus uteri; and as rupture of the perineum presupposes a diminished force of this muscle, this condition also frequently results in this displacement. Therefore,

I. The *predisposing* causes of prolapsus (and these alone may frequently produce this displacement) are:

1. *Agencies which weaken the natural supports of the uterus;* and first of all, parturition and its consequent relaxation of the parts concerned; abortion; the process of menstruation; anæmia, and its resulting general debility. A short, straight vagina, and a relaxation of this canal from disease, may also be added under this head.

2. *Agencies increasing the weight or changing the direction of the uterus.* (1.) Dr. Bennett says, a heavy, swollen cervix may even produce prolapsus. Hence congestion or hypertrophy of the uterus itself; polypus uteri, fibrous tumors, scirrhus, and arrested involution after parturition. (2.) Pressure from extra-uterine tumors or enlarged contiguous organs, especially ascites, and ovarian diseases.

II. The *exciting* causes of prolapsus include several of the agencies already enunciated as predisposing causes. The general and local relaxation from uterine or other profuse hæmorrhages, menorrhagia, or prolonged and profuse leucorrhœa, may directly produce prolapsus; or any of the before-mentioned agencies predisposing thereto, a sudden effort, as in dancing, leaping, lifting, violent laughter, coughing, vomiting, or straining in defecation, may directly force the womb downward to a point, whence it does not again rise to its natural position. Certain occupations may, therefore, act as exciting causes, as that of market-women who carry heavy burdens.

This displacement will be concluded in the next lecture.

## Figures and Tables

**Fig. 3. f1:**
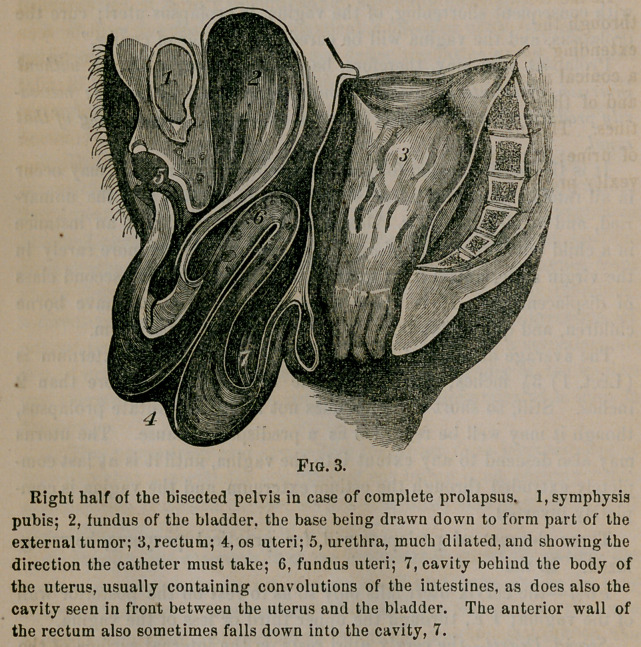


**Fig. 4. f2:**